# Two distinct variants of simian foamy virus in naturally infected mandrills (*Mandrillus sphinx) *and cross-species transmission to humans

**DOI:** 10.1186/1742-4690-7-105

**Published:** 2010-12-14

**Authors:** Augustin Mouinga-Ondémé, Edouard Betsem, Mélanie Caron, Maria Makuwa, Bettina Sallé, Noemie Renault, Ali Saib, Paul Telfer, Preston Marx, Antoine Gessain, Mirdad Kazanji

**Affiliations:** 1 Unité de Rétrovirologie, Centre International de Recherches Médicales de Franceville, Franceville, Gabon; 2 Unité d'Epidémiologie et Physiopathologie des Virus Oncogènes, URA CNRS 3015, Institut Pasteur, Paris, France; 3 Centre de Primatologie, Centre International de Recherches Médicales de Franceville, Franceville, Gabon; 4 CNRS UMR7212, INSERM U944, Institut Universitaire d'Hématologie, Conservatoire National des Arts et Métiers, Paris, France; 5 Tulane National Primate Research Center, Covington, Louisiana, USA; 6 Réseau International des Instituts Pasteur, Institut Pasteur, Paris

## Abstract

**Background:**

Each of the pathogenic human retroviruses (HIV-1/2 and HTLV-1) has a nonhuman primate counterpart, and the presence of these retroviruses in humans results from interspecies transmission. The passage of another simian retrovirus, simian foamy virus (SFV), from apes or monkeys to humans has been reported. *Mandrillus sphinx*, a monkey species living in central Africa, is naturally infected with SFV. We evaluated the natural history of the virus in a free-ranging colony of mandrills and investigated possible transmission of mandrill SFV to humans.

**Results:**

We studied 84 semi-free-ranging captive mandrills at the Primate Centre of the Centre International de Recherches Médicales de Franceville (Gabon) and 15 wild mandrills caught in various areas of the country. The presence of SFV was also evaluated in 20 people who worked closely with mandrills and other nonhuman primates. SFV infection was determined by specific serological (Western blot) and molecular (nested PCR of the *integrase *region in the *polymerase *gene) assays. Seropositivity for SFV was found in 70/84 (83%) captive and 9/15 (60%) wild-caught mandrills and in 2/20 (10%) humans. The 425-bp SFV *integrase *fragment was detected in peripheral blood DNA from 53 captive and 8 wild-caught mandrills and in two personnel. Sequence and phylogenetic studies demonstrated the presence of two distinct strains of mandrill SFV, one clade including SFVs from mandrills living in the northern part of Gabon and the second consisting of SFV from animals living in the south. One man who had been bitten 10 years earlier by a mandrill and another bitten 22 years earlier by a macaque were found to be SFV infected, both at the Primate Centre. The second man had a sequence close to SFVmac sequences. Comparative sequence analysis of the virus from the first man and from the mandrill showed nearly identical sequences, indicating genetic stability of SFV over time.

**Conclusion:**

Our results show a high prevalence of SFV infection in a semi-free-ranging colony of mandrills, with the presence of two different strains. We also showed transmission of SFV from a mandrill and a macaque to humans.

## Introduction

Foamy viruses are members of the *Spumavirus *genus of the Retroviridae family [1]. These complex exogenous retroviruses are highly prevalent in several animal species, including nonhuman primates, felines, bovines and equines, in which they cause persistent infection [2-7]. Simian foamy virus (SFV) infection has been reported in 1-6% of people occupationally exposed to nonhuman primates in zoos, primate centres and laboratories, mainly in North America but also in Europe [8-14]. Recently, naturally acquired SFV infections were described in a group of hunters living in Cameroon, central Africa [15,16], and in people in frequent contact with various macaque species in Asia [17,18]. In Cameroon, 3.6% of people who were severely bitten and otherwise injured while hunting gorillas and chimpanzees had detectable SFV infection [16].

Foamy viruses are considered to be non-pathogenic in naturally or experimentally infected animals [10,11,16,19,20]. This apparent lack of pathogenicity strongly contrasts with the cytopathic effect seen *in vitro *in infected cell cultures, with the characteristic foamy appearance of vacuolized cells [19,21,22]. It was suggested recently that the non-pathogenicity of SFV infection in nonhuman primates *in vivo *is due to replication in a superficial cell niche of the oral mucosa [23].

In contrast to lentiviruses, such as HIV and simian immunodeficiency virus (SIV), foamy viruses show little genetic drift *in vivo *[2,24-27]. Phylogenetic analysis has shown species-specific distribution of foamy viruses, indicating long-term co-evolution with their natural hosts. Switzer et al. suggested that foamy viruses have co-speciated with Old World primates for at least 30 million years [28].

While the molecular features of foamy viruses *in vitro *have been studied extensively [19,21,22,29,30], little information is available on their epidemiological and viral characteristics *in vivo *[3,4,18,20,24-26,31]. The published epidemiological studies indicate that the seroprevalence of antibodies to SFVs in captive adult nonhuman primate populations can reach 75-100% [4,20,24]. Although several reports have been published on the prevalence of SFV in semi-free-ranging colonies and wild troops of nonhuman primates [2,17,27,32-40], the timing and modes of primary infection *in vivo*, especially *in natura*, are still poorly understood.

A semi-free-ranging colony of mandrills (*Mandrillus sphinx*) was created at the Primate Centre of the International Centre for Medical Research (CIRMF) in Gabon in 1983, and more than 140 mandrills are now housed in the Centre [41]. Mandrills are found in the wild in a restricted area of central Africa, in the tropical forests of Cameroon, Equatorial Guinea, Gabon and southern Congo [41]. It has been reported previously that mandrills are naturally infected with SIV (SIVmnd) and simian T-cell leukaemia virus (STLV-1) [41-48], but little information is available on SFV infection in mandrills. Calattini et al. reported that a small series of wild-born, wild-caught mandrills in Cameroon as well as five mandrills in the Primate Centre in Gabon were infected with SFV [3]. Furthermore, recent studies showed that interspecies transmission of SFV from mandrills to humans is possible [15,16,34].

The aim of our study was to evaluate the natural history of mandrill SFV in this free-ranging colony, including the prevalence, modes of transmission, genetic diversity and origin. We also investigated cross-species transmission of mandrill SFVs to people occupationally exposed to these animals.

## Results

### SFV is highly endemic among mandrills, and the prevalence increases significantly with age

**Figure 1:**
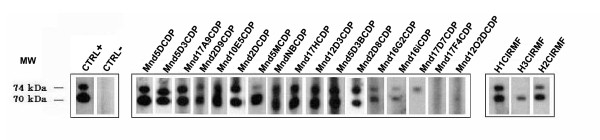
**Detection of SFV-specific antibodies by Western blot analysis in mandrill and human plasma samples**. Seropositivity was defined by the presence of reactivity to the Gag doublet of 70 kDa and 74 kDa as shown for positive controls (CTRL+). Seronegativity was defined as no bands of the gag doublet observed by Western blot, as in the negative control (CTRL-). Reactivity with a single band in the 70- to 74-kDa molecular mass range was considered indeterminate. The mandrills samples Mnd5DCP, F 19y; Mnd5D3CDP, F 11y; Mnd17A9CDP, M 4y; Mnd2D9CDP, M 6y; Mnd10E5CDP, F 5y; Mnd2DCDP, F 20y; Mnd5MCDP, M 9y; MndNB, M 5y; Mnd17HCDP, M 10y; Mnd12D3CDP, F 15y; Mnd5D3B, F 4y; Mnd2D8CDP, M 7y; Mnd16G2CDP, F 4y; Mnd16iCDP, M 8y and human H1CIRMF and H2CIRMF are seropositive. Only mandrills Mnd2DCDP, MndNB and Mnd5D3B were negative in PCR. The mandrill Mnd17D7CDP, M 4y and the human H3CIRMF are indeterminate; and mandrills Mnd17F4CDP, F 4y and Mnd12O2CDP are seronegative. Mnd: mandrill; CDP: Centre de Primatologie; in the middle: mandrill identity; M: male; F: female; Y: years. The relative molecular masses of SFVcpz-specific Gag protein are indicated on the left (MW). The Western blot positive control is a serum from an SFV-positive chimpanzee [16]. The negative serum was obtained from a person who had never been in contact with a nonhuman primate. H1CIRMF, H2CIRMF, and H3CIRMF are the results of Western blot serology for human samples.

The seroprevalence of SFV was evaluated in 84 mandrills (mean age, 8 years; range, 1-29), comprising 38 males (mean age, 7 years; range, 1-20) and 46 females (mean age, 8.6 years; range, 2-29). Of these, 28 were juveniles (< 4 years); 36 were sub-adults (5-10 years); 6 were adults (11-15 years), and 14 were old adults (> 16 years) (Table 1). We found by Western blot analysis that 70 of the 84 mandrills had *gag *doublet reactivity, and they were thus considered SFV seropositive (Figure 1), for an overall seroprevalence of 83%. Four were of indeterminate seropositivity, and the 10 others were considered seronegative. As seen in Table 1 the seroprevalence increased significantly with age (*p <*0.001), from 57% in juvenile monkeys to 94% in adults and 100% in older mandrills. No significant difference was found between males (84%) and females (82%).

**Table 1:** Seroprevalence and PCR results for SFV in semi-free-ranging mandrills, by age and sex

Age (years)			Male				Female				total	
	**No**. **positive/** **tested**	**%**	**[95% CI]**	**No**. **sequence/** **positive** **PCR**	**No**. **positive/** **tested**	**%**	**[95% CI]**	**No**. **sequence/** **positive** **PCR**	**No**. **positive/** **tested**	**%**	**[95% CI]**	**No**. **sequence/** **positive** **PCR**

1-4 (juveniles)	7/12	58	[30-86]	2/3	9/16	56	[32-90]	7/7	16/28	57	[39-75]	9/10

5-10 (young adults)	19/20	95	[86-105]	19/19	15/16	94	[83-105]	12/13	34/36	94	[86-102]	21/32

11-15 (adults)	1/1	100		0/1	5/5	100		5/5	6/6	100		5/6

> 16 (old adults)	5/5	100		0/5	9/9	100		8/8	14/14	100		8/13

Total	32/38	84	[73-95]	21/28	38/46	82	[71-93]	32/33	70/84	83	[75-91]	53/61

### Molecular detection of SFV and genetic diversity in mandrills

The DNA samples obtained from peripheral blood mononuclear cells (PBMCs) from the 84 mandrills were examined by nested PCR targeting a 425-bp fragment of *integrase*, a region in the polymerase gene. The 14 seronegative and indeterminate samples were PCR negative. SFV DNA was detected in 61 of 70 seropositive samples (87%); although the other nine mandrills were serologically positive, no SFV DNA could be detected. The sequence of the *integrase *fragment was obtained for 53 PCR-positive samples (Table 1). Nucleotide sequence comparison showed that 52/53 sequences were closely related, with 94-100% sequence similarity, and they were also closely related to the five SFV sequences previously obtained by Calattini et al. [3]. The one divergent sample, Mnd31CDP, from a wild-born mandrill introduced into the colony at the age of 2 years, showed greater nucleotide divergence (8-9%) than all the other mandrill SFV sequences.

**Figure 2:**
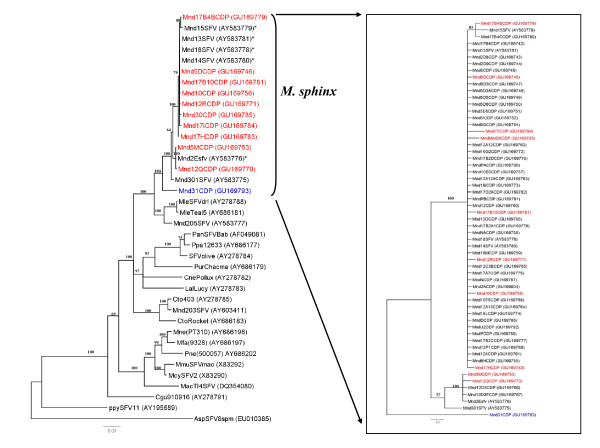
**Phylogenetic relationship of *integrase *sequences (425 bp) circulating in the mandrill colony at the CIRMF**. Phylogenetic tree of the 11 main circulating sequences (in red, mandrills harbouring virus from northern Gabon; in blue, from southern Gabon), representing all sequences in the colony. The five SFV sequences obtained previously by Calattini et al. [3] are identified with an asterisk. The insert shows all 53 sequences, including clone 11 (in colour). All SFV sequences were aligned with ClustalW (1.81) and edited with Bioedit. Phylogenetic analyses were performed with the Bayesian Markov chain Monte Carlo (BMCMC) method implemented in MrBayes 3.1 and the Rtrev model. Sequence AspSFV8spm (from a New World spider monkey) was included as an outgroup. The maximum clade credibility tree topology inferred with FigTree v1.2 is shown. Values above the branches are bootstrap values. All new mandrill sequences are identified by Mnd (for mandrill), a number (frequently followed by a letter) and ending with CDP (Centre de Primatologie, their origin) (vg: Mnd12QCDP). In brackets is the accession number in GenBank.

The phylogenetic analysis confirmed these findings, as shown in Figure 2. This tree represents the 11 main SFV strains circulating in the colony and, in the insert, all 53 sequences, including the 11 main strains (in colour). These 53 newly obtained SFV strains belong to a large clade comprising all the available sequences from mandrills and drills, with a high bootstrap value (100%). This clade contains two main clusters. The first comprises most of the new sequences and others previously obtained from mandrills, including the five sequences of Calattini et al. [3], from the same breeding centre. The second consists of the unique Mnd31CDP strain, which is localized between the large clade of mandrills and that of drills (Figure 2).

### Mandrills in Gabon are naturally infected with two distinct variants of simian foamy virus

**Figure 3:**
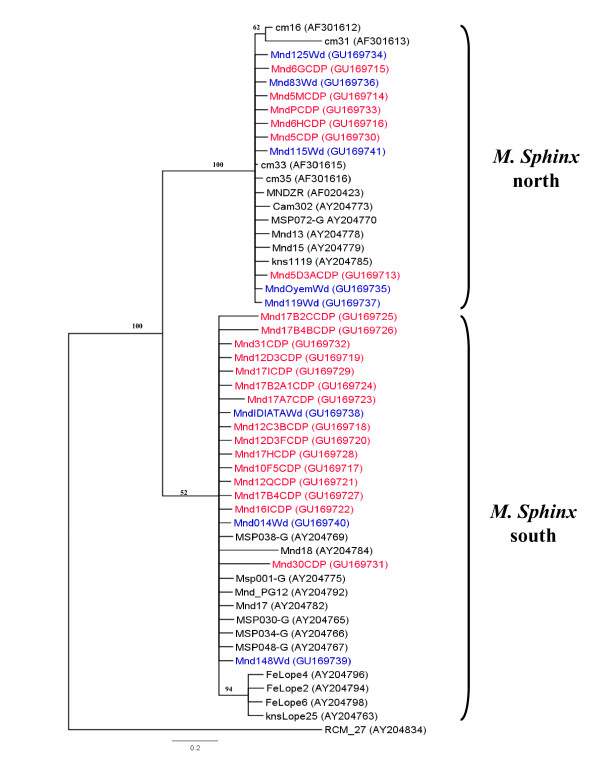
**Phylogenetic tree from 267 bp of the mitochondrial cytochrome b gene from some of the mandrills in the CIRMF colony**. Phylogenetic tree of sequences from 21 mandrills in the colony at the CIRMF (in red) and 8 wild mandrills (in blue) inferred as described in Figure 2. Wild mandrills are indicated as Mnd (for mandrill), a number or a name and Wd (for wild) (vg:Mnd125Wd). An outgroup was a sequence of RCM_27 (from a red-capped mangabey).

**Figure 4:**
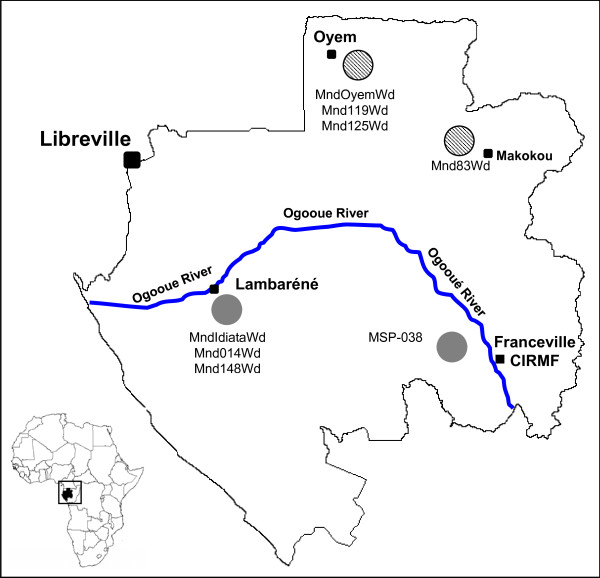
**Location of SFV-positive wild mandrills**. Map of Gabon, with the capital (Libreville) and main cities (Oyem, Lambaréné, Makokou, and Franceville) and locations of the samples collected from SFV-positive wild mandrills (Mnd and MSP). Line in blue represents the Ogooué River, which divides the country.

To determine the origin and distribution of the different clades in the mandrill colony, a 267-bp portion of the cytochrome b sequence was amplified and sequenced from 21 SFV-infected monkeys in the colony and from eight mandrills caught in the wild (Figure 3) in various regions of Gabon (Figure 4).

As seen in the phylogenetic tree, two distinct clusters could be distinguished, with perfect correlation between cytochrome b sequences and the origin of the wild mandrills. One cluster consisted of mandrills from regions north of the Ogooué River and the second of animals from regions south of the Ogooué. The Mnd31CDP cytochrome b sequence clustered with sequences obtained from mandrills originating in southern Gabon, as did 14 of 21 analysed sequences of cytochrome b from our colony. Only six sequences from other mandrills in our colony clustered with sequences from mandrills from northern Gabon (above the Ogooué River, see Figure 4).

**Figure 5:**
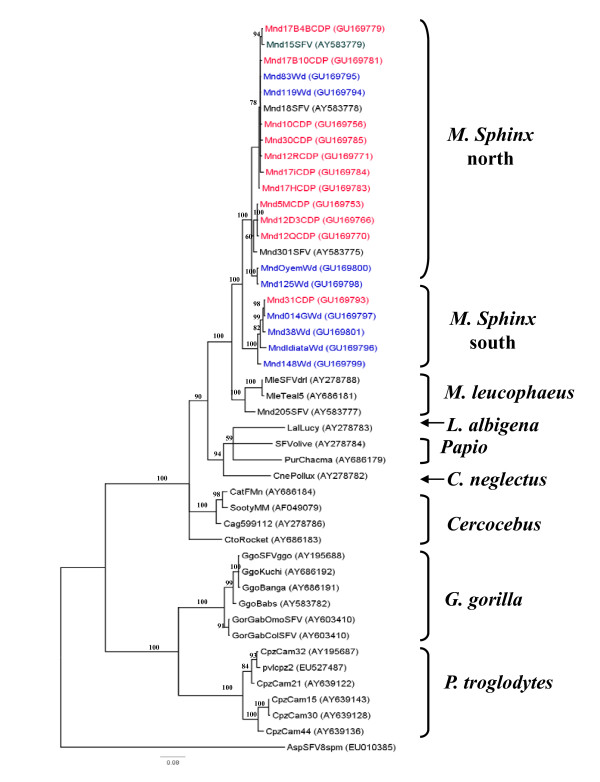
**Phylogenetic confirmation of the presence of two circulating SFV strains among mandrills**. Phylogenetic tree of the 425-bp fragments of a region of the *integrase *region in the SFV polymerase gene. All 11 representative strains newly identified from mandrills in the colony (in red) at the CIRMF and the 8 new strains identified from wild mandrills (in blue) located in various regions of the country are shown in the tree. The new strains from mandrills were analysed with SFV sequences obtained from various species of nonhuman primates available in Genbank. The phylogenetic tree was obtained by the Bayesian method implemented in MrBayes version 3.1 software as described in the legend to Figure 2. The names of the different nonhuman primate species included in the tree are listed on the right side of the tree.

To confirm the hypothesis that mandrills are infected naturally with two different SFV strains, we amplified and sequenced SFV from DNA in blood or tissue samples collected from eight mandrills (pets or 'bush meat') from northern Gabon and seven from the southern part (Figure 4). Eight SFV sequences were obtained and compared with the SFV in our colony. Phylogenetic analysis confirmed that mandrills are infected with two SFV strains (Figure 5). Mnd31CDP clustered with the SFV obtained from wild monkeys from the south, whereas the other strain clustered with newly obtained viruses from wild northern animals. Cytochrome b phylogenetic analysis also confirmed the geographical separation of the wild mandrills (Figure 3).

### Transmission of SFV from mandrills to humans

**Figure 6:**
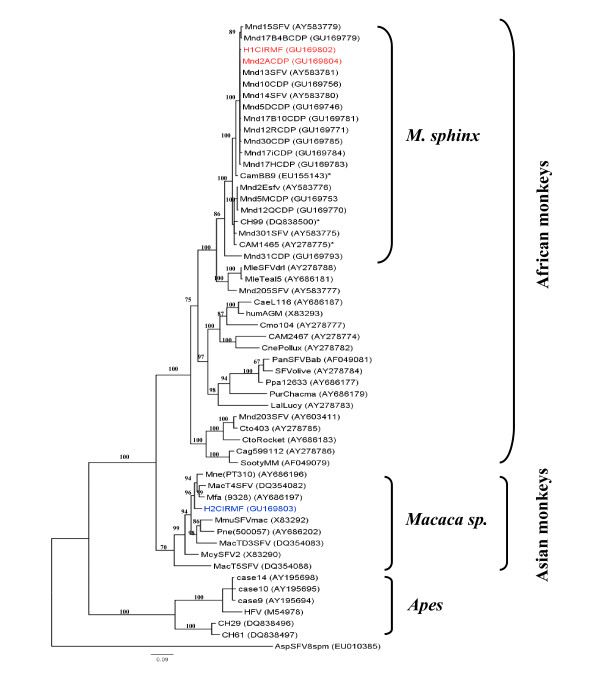
**Phylogenetic tree of the 425-bp fragments of the SFV *integrase *sequences obtained from two workers at the Primate Centre of the CIRMF**. The two cases of SFV infection are in colour: red for the first and blue for the second. The origin of the first SFV sequence (H1CIRMF) is clearly defined as a mandrill (Mnd2ACDP), shown in the same colour. The second SFV sequence (H2CIRMF) clusters with Asian macaque sequences. The tree was inferred as described in Figure 2. Identified by an asterisk are the three published mandrill sequences known to infect humans. Human sequences are indicated by H (for human), a number (1 or 2) and CIRMF (Centre International de recherches Médicales de Franceville), where the study was performed.

We evaluated the possible transmission of mandrill SFV to humans by examining 20 people (15 men and 5 women; mean age, 39 years; range, 20-54) occupationally exposed to mandrills as animal caretakers or veterinarians at the Primatology Centre. The mean duration of exposure to nonhuman primates was 12 years (range, 5 months to 27 years). Two of these people (10%) were found to be SFV-seropositive by Western blotting (Figure 1). The SFV *integrase *sequence was detected by nested PCR in PBMCs from the two seropositive persons, who were found to be the only ones who had been bitten by nonhuman primates during their work at the Centre. The first person (H1CIRMF) was bitten by a chimpanzee on a finger in 1996 and by a mandrill (Mnd2ACDP) on a shoulder during the same year. The second person (H2CIRMF) recalled a bite on a finger by an unknown monkey in 1985. SFV sequences were obtained from amplified 425-bp *integrase *fragments in PBMC DNA from the two SFV seropositive persons as well as from the chimpanzee and the mandrill Mnd2ACDP. Phylogenetic analysis (Figure 6) showed that the viruses from H1CIRMF and from mandrill Mnd2ACDP were almost identical, with only one base difference (99.7% nucleotide identity). This sequence was not related to the sequence obtained from the chimpanzee. Phylogenetic analysis of the SFV obtained from the second person showed that the virus was located in the clade of Asian SFVs (bootstrap, 96%) and clustered with *Macaca fascicularis *(Figure 6). The two SFV-infected humans are healthy and show no clinical signs related to a retroviral infection, 15 years after the bites.

### SFV shows extremely low genetic drift in mandrills and humans

To evaluate the genetic variability of SFV *in vivo*, we investigated the virus population in one mandrill at an interval of 10 years, and we also studied the genetic variation of the virus after transmission to a human through a severe bite. We studied several clones obtained in a single PCR: 18 clones from mandrill Mnd2ACDP in 1996 on the day H1CIRMF was bitten, 13 clones from the same animal 10 years later, and 11 clones from the bitten person 10 years after the bite. Comparative sequence analysis showed strong nucleotide sequence similarity (data not shown), with a major identical strain (12/18 and 9/13 clones identical) among the sequences obtained in the mandrill on day 0 and 10 years later. The major strain (4/11 clones) in the infected person differed by one base from the major mandrill strain. The other clones in the two mandrill and the human samples differed only slightly, with a divergence of one or two bases. Also, as seen in Additional file 1 clones sequenced from H1CIRMF clustered mainly at the top of the tree, while sequences of the clones from Mnd2ACDP clustered in the middle, close to some published sequences. Only one clone sequence from H1CIRMF, CIRMF1C9, was closely related to a clone sequence from Mnd2ACDP (Mnd2AC10Y10).

## Discussion

We found a high seroprevalence of SFV in a semi-free-ranging colony of mandrills originating from and living in Gabon, central Africa. The habitat of mandrills is restricted to western central Africa, which is highly endemic for other retroviruses, such as SIV and STLV [42-47]. A seroprevalence of 89.5% was found in a small free-ranging macaque population (mostly adults) living in a temple in Bali, Indonesia, with a higher prevalence in adults than in juveniles [18,31,39]. A larger study provided evidence that *Macaca tonkeana *acquire SFV mainly through severe bites, mainly when young adults aged 5-8 years compete for sex partners [27]. In a study of free-ranging colonies of chimpanzees, Liu et al. found a significant increase in SFV infection with age, with no evidence of vertical transmission to the young [32]. In our study, there was a clear increase in SFV infection at 4-5 years of age. Altogether, these findings indicate horizontal rather than vertical (perinatal) transmission as the predominant route of SFV infection in these nonhuman primate communities. Nevertheless, some species or colony specificity may be found *in natura *among troops of nonhuman primates, which might change the relative importance of different modes and thus the timing of SFV transmission.

It is known that a similar virus can be transmitted quite differently in different nonhuman primate species: STLV-1 appears to be acquired mainly in breast milk in *M. tonkeana *[27] but is acquired mainly in adulthood in chimpanzees [18,33,49]; in mandrills, it is probably acquired through bites [42,46-48,50] and to a lesser extent by sexual contact, and a predator-prey system may sometimes be also involved [49]. In our mandrill colony, about 50 animals were SFV-positive at the age of 1 year, perhaps due to exchange of saliva with their mother during feeding. It was reported recently that mandrills have a prominent muzzle-muzzle behaviour, usually between young naive and older individuals [51,34-44]. It has also been reported that salivary glands are the major reservoir of SFV replication in monkeys [23,26,29]. We did not observe any difference in seroprevalence according to the sex of the animals. SFV seroprevalence increased significantly with age. These findings are similar to those on the seroprevalence of STLV-1 in this colony, which was evaluated at 13.4% [52].

Our study indicates that all except one *integrase *sequence of the SFV strains circulating in the colony are closely related, and some are identical. The probable explanation is related to the history of the colony, which was founded in 1983 with only a few animals, some of which probably harboured a virus originating from northern Gabon. The virus was therefore transmitted and spread in the colony during the past 25 years by the founders from the northern part of the country. Ten different strains are circulating in the northern group, with 96-99% sequence similarity. Similar observations have been made with regard to the circulation of several strains in other nonhuman primates, including monkeys and apes [2,17,27,53].

The animal that harboured the eleventh strain circulating in the colony, which is quite different from the other strains, was a wild-born mandrill brought to the Primate Centre in 2003 from the southern part of the country at the age of 2 years. It was kept in quarantine for 6 months and then introduced into the mandrill colony. Dissemination of the virus could occur in several ways, as indicated above, but also because one of the infected mandrills is a dominant male in the colony. This hypothesis cannot, however, be confirmed, since no sample was available from the first mandrills introduced into the colony.

Our finding that two different strains exist in the colony suggests that mandrills living currently in northern and southern Gabon are infected by two different SFV strains. Similar situations have been reported for two other retroviruses that infect these monkey species, SIV [43] and STLV-1 [47]. As seen in Figure 3 the cytochrome b study showed that most of the mandrills are from the south but are infected with a SFV strain from the north. This suggests that they were infected in the breeding colony by a SFV virus from a mandrill originating from the north (Figure 2), except for mandrill 31 (see above). In contrast, the origin of each wild mandrill (Figure 3) was concordant with the virus they harboured (Figure 5), confirming infection in their natural area. Furthermore, studies of cytochrome b polymorphism suggest that the Ogooué River separates mandrill populations into two different phylogenetic groups: one in the north (northern Gabon and Cameroon) and the other south of the River (southern Gabon and Congo) [54].

Monkeys have a long co-existence with their SFV [2,24,28,32,33,53,55,56], which would have started when mandrills in both the north and the south had a common ancestor and has persisted since their separation, about 800 000 years ago [54]. These results for SFV infection in mandrills are supported by the fact that the same mandrills are infected with SIV [43] and STLV [47]. Our analysis of the results for 15 wild mandrills caught in the northern and southern parts of Gabon clearly indicates the existence of two different variant strains of SFV. The discrepancy in our study between serological data and the absence of the SFV sequence in mandrill PBMCs may be due to a low viral load in blood samples. In some juveniles, it could be the result of high levels of maternal antibodies against SFV [2].

We also found that two of 20 people working at the Primate Centre were infected with SFVs: one with a mandrill strain and the second with a macaque virus. Only about 50 people worldwide have been shown to be SFV-infected (both serologically and molecularly) [13,14], including people occupationally exposed to nonhuman primates [12,25] and people at risk in natural settings, such as hunters in central Africa [15,16]. Furthermore, only three other human infections with mandrill SFV have been reported. In the first case, a hunter living in Cameroon was found to be infected by a mandrill strain, but the route of infection was not documented [15]. The second case was in a blood donor in Cameroon, also with no information on the route of infection [34]. In the third case, a man aged 26 years had been bitten by a small monkey while hunting 1 year before the presence of mandrill SFV was found [16]. We demonstrated the identity of the viral foamy strain in the donor (Mnd2ACDP) by molecular sequencing at the time of the bite that probably transmitted the virus, and in the human recipient 10 years later, with 99% similarity between the two sequences. This person had been bitten only once by mandrill Mnd2ACDP and not by other mandrills. The presence of a sequence from the clones of H1CIRMF (CIRMF1C9) among clone sequences from Mnd2ACDP, particularly Mnd2AC10Y0 (Additional file 1), sustains the hypothesis of the origin of H1CIRMF virus from Mnd2ACDP. No close sequence similarity was found between the H1CIRMF sequence and the three other sequences previously found in humans infected by a mandrill SFV [15,16,34] (Figure 6).

Only one molecular demonstration of SFV interspecies transmission has previously been reported, due to a bite by a chimpanzee to a zoo worker [12]. Although the person infected by the mandrill virus in our study had also been bitten during his professional activity by a chimpanzee, we were unable to detect any chimpanzee SFV sequence in his PBMCs. 'Dual' risks with only one virus detectable by PCR have also been reported in hunters in south Cameroon [16]. Co-infection with two different simian viruses was demonstrated recently in chimpanzees infected not only with their own chimpanzee SFV, but also with a Colobus strain [49]. The second human was infected with a strain related to a macaque SFV. Despite the use of thousands of macaques in biomedical research, primate facilities and institutions for decades (in both Europe and North America), only one case of human infection with a macaque foamy virus has been reported (in a worker in Canada after a severe bite) [9]. In contrast, recent studies in Asia showed transmission of macaque SFV to nine people, including zoo workers, owners of nonhuman primate pets, 'bush meat' hunters and temple workers [17,18]. Mathematical modeling shows that, in Bali, about six of every 1000 visitors to monkey temples will be infected with SFV [39].

In our work, we also observed high stability of the *integrase *sequence of SFV over time (10 years in an infected mandrill as well as in an infected human), with neither genetic drift over time nor the presence of quasi-species. Foamy viruses are genetically very stable [57] and, with the exception of cross-species transmissions, have co-evolved with their hosts [28]. Their high genome conservation often allows attribution to a particular monkey or ape subspecies through analysis of the appropriate foamy virus sequence [27,32,33]. Furthermore, in cross-species transmission to humans or apes, the transmitted virus can be easily traced back to the transmitting monkey species and appears to be genetically stable in the new host for decades [53,58,59].

In conclusion, we have shown that SFV is highly endemic in mandrills in Gabon, and this virus can be transmitted to humans. Further studies are being conducted to evaluate the prevalence of this virus in larger samples from various monkey species in central Africa. We are also studying the natural transmission of these viruses to human populations living in this geographical area, where consumption of 'bush meat' and hunting are common.

## Materials and methods

### Mandrills and biological samples

We studied 84 mandrills in the semi-free-ranging colony housed at the Primatology Centre of the International Centre for Medical Research in Franceville, Gabon. Wild-born, wild-caught animals and animals born at the Centre were maintained in accordance with the guidelines of the United States National Institutes of Health. The six male and eight female mandrills that founded the colony were brought from various parts of Gabon and released into the enclosure in 1983 [43,46]. A small colony of macaques (*M. fascicularis*) was also founded in 1983. Blood samples from monkeys in this colony are collected every year, stored at -80°C and tested for different retroviruses, including SFV. Thus, between November 2006 and January 2007, 7 ml of blood were collected from mandrills in EDTA-K2 tubes under ketamine HCl anaesthesia (10 mg/kg body weight). Plasma and PBMCs obtained after Ficoll separation were kept frozen.

Wild mandrills were collected in cities and villages throughout the country and in the Lopé Reserve. We collected blood samples from locally captured live animals (pets) and from wild mandrills as previously described [43]. Small amounts of tissue (donated by hunters) were also collected from fresh cadavers in villages or on markets [54]. No money or favours were exchanged for these samples in order to prevent any increase in demand for 'bush meat'. All samples were collected with the approval of the Gabonese Government and in accordance with national laws. Tissue samples were immediately preserved and then stored at -20°C until tested.

To evaluate possible transmission of SFV from mandrills to humans, blood was collected from caretakers or veterinarians working at the Primatology Centre. The participants were volunteers, and fully informed consent was obtained from each person before testing. The samples were anonymous, but age and information about the contact, such as a bite, scratches or other wounds, were retained (for 12 years of mean length of potential exposure to animals). The study obtained ethical clearance from the public health authorities.

### Serological studies

Plasma from mandrills was screened for the presence of foamy virus antibodies as described previously [4,25,60]. Briefly, a Western blot assay was performed with an SFV-infected BHK-21 cell line as the source of foamy viral antigens [27]. Plasma was tested at 1:100 dilution. Western blot seropositivity was defined as the presence of reactivity to the Gag doublet of 70 kDa and 74 kDa, as previously described [4]. Samples without reactivity to either Gag protein were considered seronegative, and those with reactivity to a single band in the 70- to 74-kDa molecular mass range were considered indeterminate. The Western blot positive control was serum from an SFV-positive chimpanzee, used by Calattini et al. [16]. The negative serum was obtained from a human who had never been in contact with a nonhuman primate.

### Molecular studies

High relative molecular mass genomic DNA was extracted from PBMCs from the tested animals and tested against several positive and negative controls with the Qiagen kit (QIAmp blood Mini Kit, Courtaboeuf, France). The first round of PCR involved a described set of primers [61] (primer 1: GCC ACC CAA GGG AGT TAT GTG G, and primer 2: GCT GCA CCC TGA TCA GAG TG) for amplifying an *integrase *fragment of 590 bp (a region in the *polymerase *gene), under the following conditions: 40 cycles of 30 s of denaturation at 94°C, 30 s of annealing at 55°C and 1 min of extension at 72°C. A 425-bp fragment corresponding to another portion of the *integrase *was amplified under the same conditions with nested primers (primer 3: CCT GGA TGC AGA GTT GGA TC and primer 4: GAA GGA GCC TTA GTG GGG TA), as reported previously [25,60,61].

The presence and quality of the extracted DNA were verified by amplifying an *albumin *gene fragment. Amplification and detection of albumin were carried out as described for SFV pol sequences, but with specific primers (forward: AlbF: GCT GTC ATC TCT TGT GGG CTG T and reverse: AlbR: ACT CAT GGG AGC TGC TGG TTC) [62]. Molecular amplification was also performed, with the same program, to study the 267-bp cytochrome b region, which was sufficiently variable to differentiate the northern and southern populations of mandrills [54] with these specific primers: L14725: CGA AGC TTG ATATGA AAA ACC ATC GTT G and H15149: AAA CTG CAG CCCCTC AGA ATG ATA TTT GTC CTC A [63]. Positive PCR products were directly sequenced. In order to evaluate genetic drift *in vivo*, purified PCR products were cloned with the pCR2.1 TOPO plasmid (Invitrogen, Carlsbad, California, USA), and various positive clones were selected, extracted, purified and sequenced with an automatic sequencing system (GATC, Germany).

### Nucleotide sequence accession numbers

All the SFV and cytochrome b sequences from mandrills and humans obtained in this study have been submitted to GenBank as cytochrome b (accession numbers GU169713 to GU169741) and SFV (accession numbers GU169742 to GU169847).

### Phylogenetic analysis

For the phylogenetic analysis, the new SFV sequences were aligned with the ClustalW (1.81) program [64] and then analysed and edited with Bioedit http://www.mbio.ncsu.edu/BioEdit/bioedit.html. The final alignment was submitted to the the Bayesian method implemented in MrBayes version 3.1 software (2005) [65] with the Jones, Taylor and Thornton model [66] and the rtREV model [67] of evolution and gamma distributed rates at sites, with one million generations and burn-in of 2.5%. Bayesian parameters were examined with the Tracer program http://evolve.zoo.ox.ac.uk/Evolve/Software.html to determine convergence to a stable log likelihood value. Likelihood traces between replicate runs were compared for convergence to similar log likelihood values. All estimated sample sizes were greater than 545 [68]. If replicate runs converged, all trees after burn-in were combined to create a single consensus tree. BMCMC posterior probability values represent the proportion of MCMC samples that contain a particular node. The final phylogenetic tree was obtained by majority rule consensus and after editing with the graphic resources contained in the FigTree v1.2 software http://tree.bio.ed.ac.uk/software/figtree.

### Statistical analysis

SFV serological status in relation to sex and age group was analysed statistically by the chi-squared test with Yates correction, and prevalence and odds ratios were calculated. The corresponding 95% confidence intervals were reported as measures of statistical significance. The Mann-Whitney U test was also used for statistical analysis. Significance was assumed at *p *< 0.05. All analyses were performed with Statistica software v7.1 (StatSoft France, http://www.statsoft.fr).

## Competing interests

The authors declare that they have no competing interests.

## Authors' contributions

MK and AG conceived and designed the study; AMO, EB and MC performed the experiments; AMO, MC, MM, AG and MK analysed the data and wrote the paper. AS, PT, PM and BS contributed reagents and materials. All the authors were involved in drafting the paper.

## Supplementary Material

Additional file 1 **Figure S. Phylogenetic tree of all clones from H1CIRMF and Mnd2ACDP**. SFV clones of 425 bp of *integrase *fragments obtained from H1CIRMF (in red) were aligned with those from Mnd2ACDP (in blue). Phylogenetic analyses were done as described in the legend to Figure 2. Clones from H1CIRMF were identified as CIRMF (see Figure 6), a number, C (for clone) and another number (vg: CIRMF1C9). Clones from Mnd2ACDP are in two groups: on the day of injury: Mnd2A (the mandrill), followed by C (for clone, with a corresponding number) and ending with J0 (day of injury). The clones obtained 10 years after the injury have Y10 (10 years after) at the end. An outgroup is the sequence Mnd203SFV (reported by Calattini et al. [3] as originating from a drill, but clustering with *Cercocebus *species). Click here for file
