# Supplementary figures and images for: Two distinct variants of simian foamy virus in naturally infected mandrills (Mandrillus sphinx) and cross-species transmission to humans
Source: Retrovirology. 2010 Dec 14;7:105. doi: 10.1186/1742-4690-7-105 (PMC3009703; doi:10.1186/1742-4690-7-105)

## Slide 1
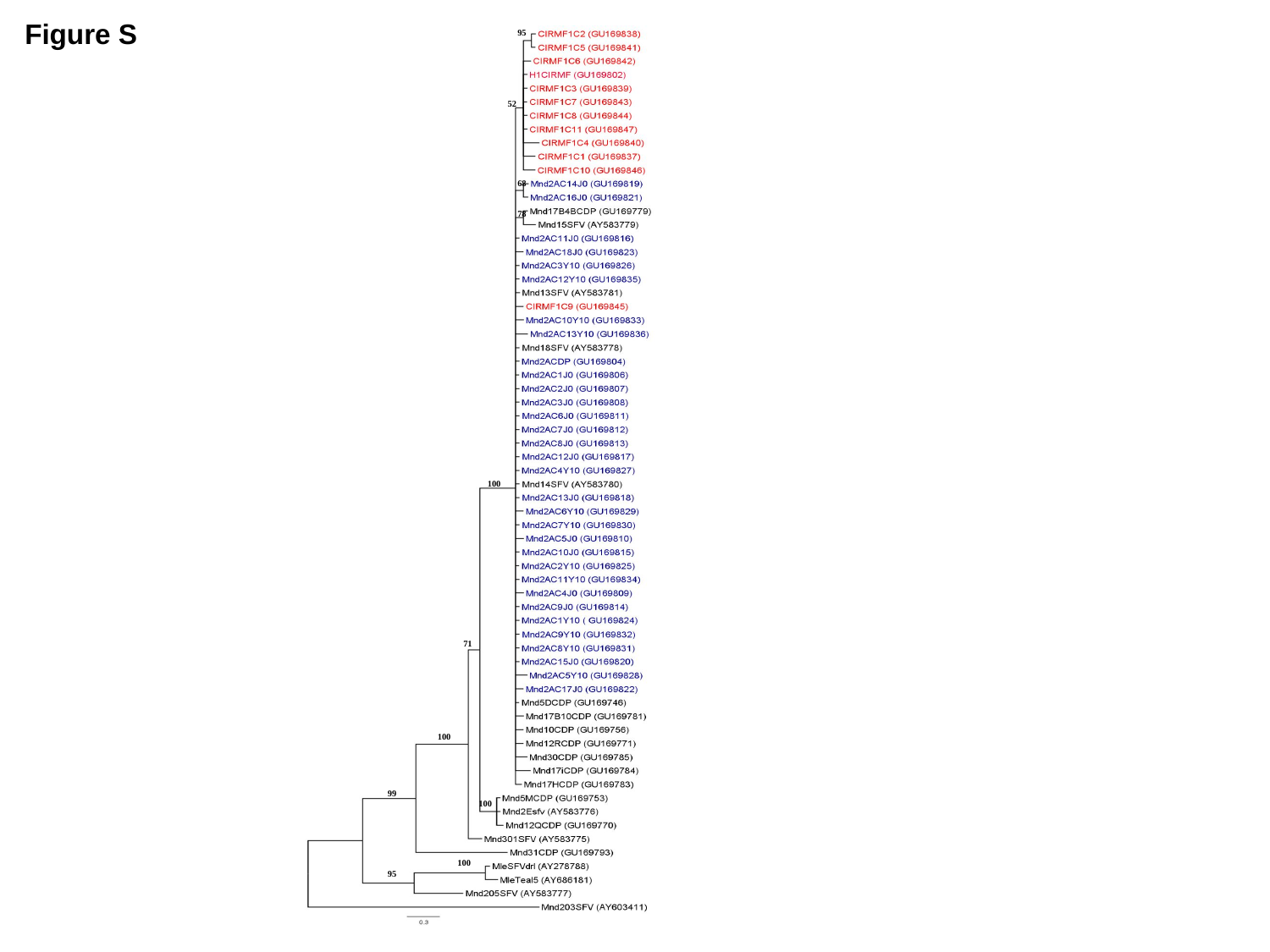

Figure S
95
52
68
78
100
71
100
99
100
100
95

Supplement: Additional file 1 — Figure S. Phylogenetic tree of all clones from H1CIRMF and Mnd2ACDP. SFV clones of 425 bp of integrase fragments obtained from H1CIRMF (in red) were aligned with those from Mnd2ACDP (in blue). Phylogenetic analyses were done as described in the legend to Figure 2. Clones from H1CIRMF were identified as CIRMF (see Figure 6), a number, C (for clone) and another number (vg: CIRMF1C9). Clones from Mnd2ACDP are in two groups: on the day of injury: Mnd2A (the mandrill), followed by C (for clone, with a corresponding number) and ending with J0 (day of injury). The clones obtained 10 years after the injury have Y10 (10 years after) at the end. An outgroup is the sequence Mnd203SFV (reported by Calattini et al. [3] as originating from a drill, but clustering with Cercocebus species). [file 1742-4690-7-105-S1.PPT]
